# Ocular Inflammatory Myofibroblastic Tumor in the Left Eye with Phthisis Right Eye: A Rare Occurrence in a Child

**DOI:** 10.1155/2015/281528

**Published:** 2015-10-18

**Authors:** Sangeeta Shah, Badri Prasad Badhu, Poonam Lavaju, Anju Pradhan

**Affiliations:** ^1^Department of Ophthalmology, Bisheshwar Prasad Koirala Institute of Health Sciences (BPKIHS), P.O. Box 7053, Kathmandu, Dharan, Nepal; ^2^Department of Pathology, Bisheshwar Prasad Koirala Institute of Health Sciences (BPKIHS), P.O. Box 7053, Kathmandu, Dharan, Nepal

## Abstract

Inflammatory myofibroblastic tumor (IMT) is a benign pseudoneoplastic inflammatory condition with the potential for persistent local growth and recurrence that rarely affects the orbit. We report a very rare case of anterior orbital IMT in a child who presented with gradually progressive mass in left eye for 16 months. Ocular examination showed a cauliflower like exophytic mass at 360 degrees of the perilimbal area covering the entire cornea and obscuring the visualization of anterior and posterior segments. The right eye was phthisical. CT scan showed a lobulated exophytic soft tissue mass in the preseptal region and along the anterior portion of the left globe extending from medial canthus to the lateral canthus. Enucleation of the left eye was performed and the histopathological examination confirmed the diagnosis of IMT. This report aims to raise awareness about this rare ocular entity and emphasizes its early treatment as delay can result in loss of the eye.

## 1. Introduction

Inflammatory myofibroblastic tumor (IMT) is a benign pseudoneoplastic inflammatory condition of unknown etiology and diverse morphology, also referred to as pseudosarcomatous myofibroblastic proliferation, xanthomatous pseudotumor, and plasma-cell granuloma [1].

IMT was originally described in lungs and other sites such as abdomen (mesentery), retroperitoneum, and genitourinary tract. Ocular involvement has rarely been reported [2]. It has the potential for local growth and recurrence [3]. In rare cases, there may be a low grade sarcomatous transformation [3]. Fortunately, the prognosis is good following surgical resection [4].

We are reporting this case because of its rare presentation involving the eyes and the challenges involved in diagnosis.

## 2. Case Report

**Figure 1:**
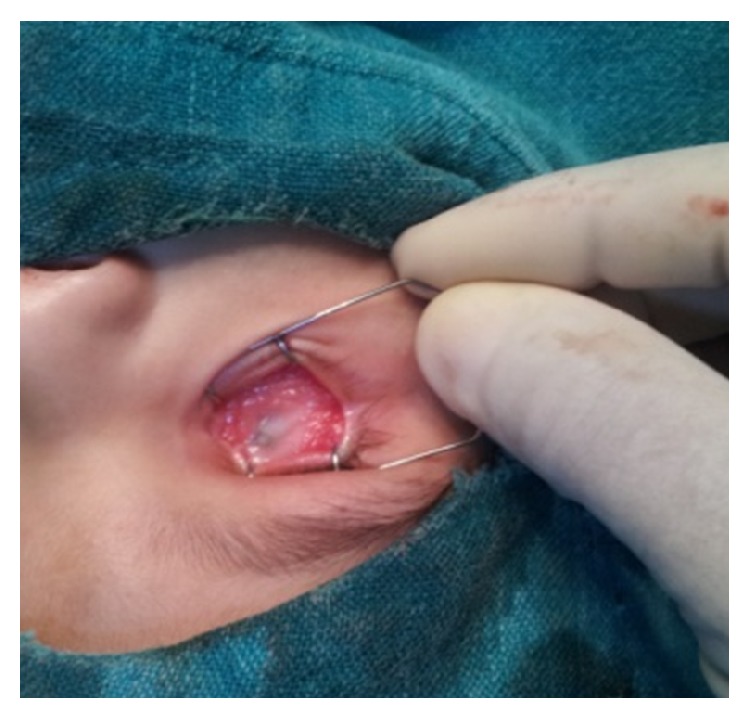
Phthisical right eye.

**Figure 2:**
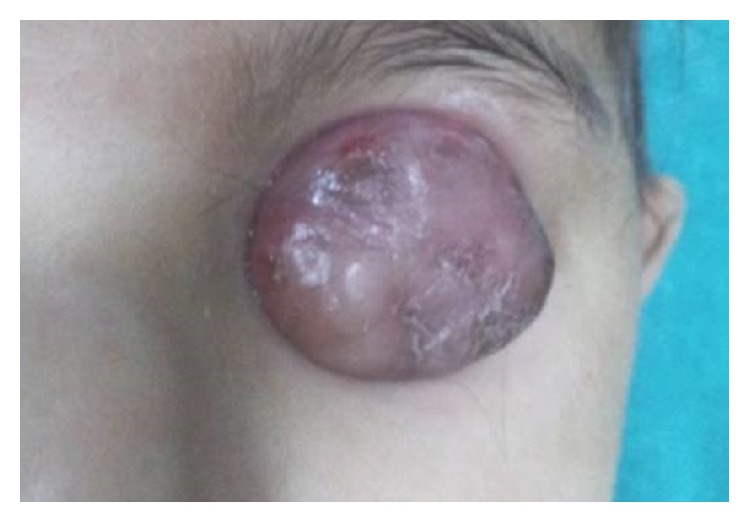
Cauliflower like mass in the left eye.

An eighteen-month-old boy was brought with the complaint of a gradually progressive painless mass in the left eye since 16 months. According to the parents, the child was unable to see with both the eyes since birth. General and systemic examination of the child was normal. Ocular examination revealed phthisical right eye (Figure 1) and a cauliflower like mass arising from the limbus (360 degrees) in the left eye (Figure 2). The entire anterior surface of the left eye was covered by the mass, obscuring the view of anterior and posterior segments. Engorged, tortuous conjunctival feeder vessels were found surrounding the mass.

**Figure 3:**
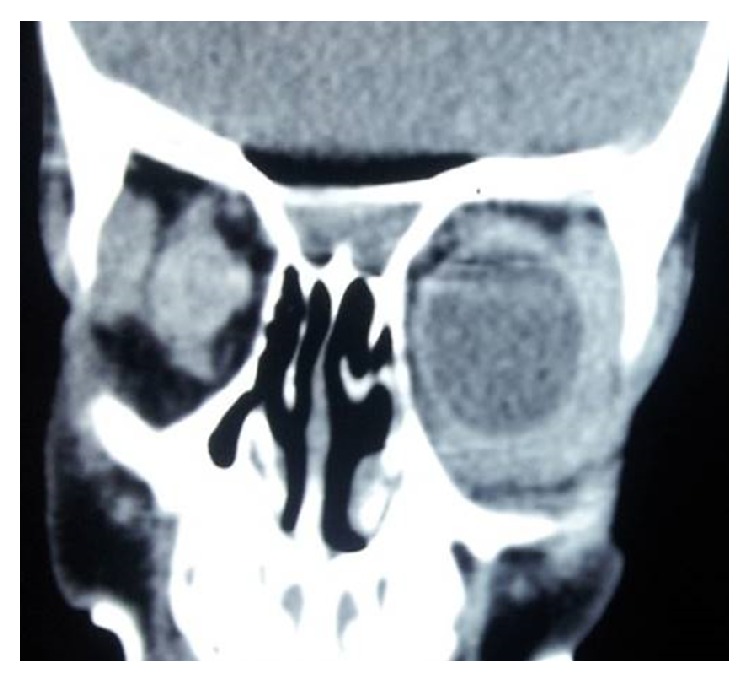
Right phthisical eye.

**Figure 4:**
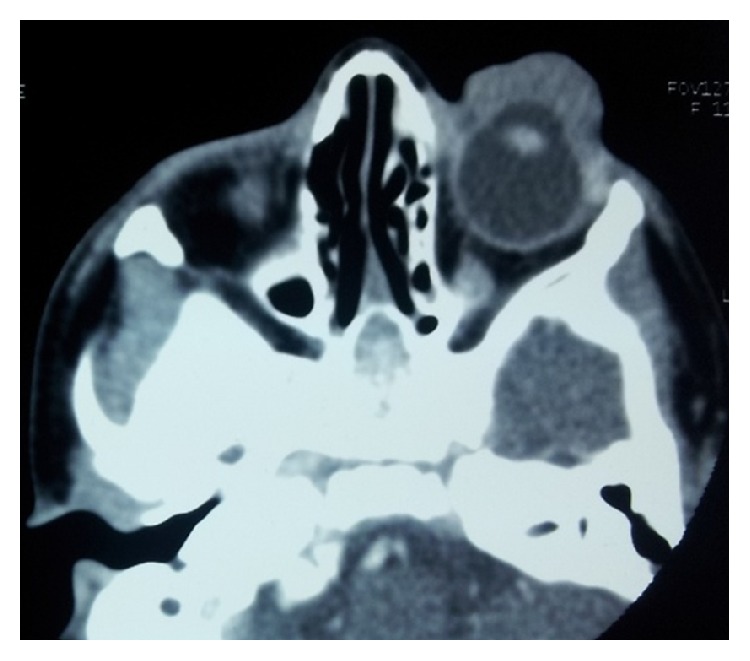
Lobulated soft tissue mass covering the anterior segment of left eye.

USG B scan showed unidentifiable phthisical right eye and normal posterior segment of the left eye. CT scan of the orbit showed right eye phthisis (Figure 3) and a lobulated soft tissue mass measuring 27 × 7 mm^2^ in preseptal region extending from lateral canthus to medial canthus of the left eye abutting the anterior globe and lacrimal gland with loss of intervening fat plane (Figure 4). The mass was completely covering anterior segment of the left globe.

**Figure 5:**
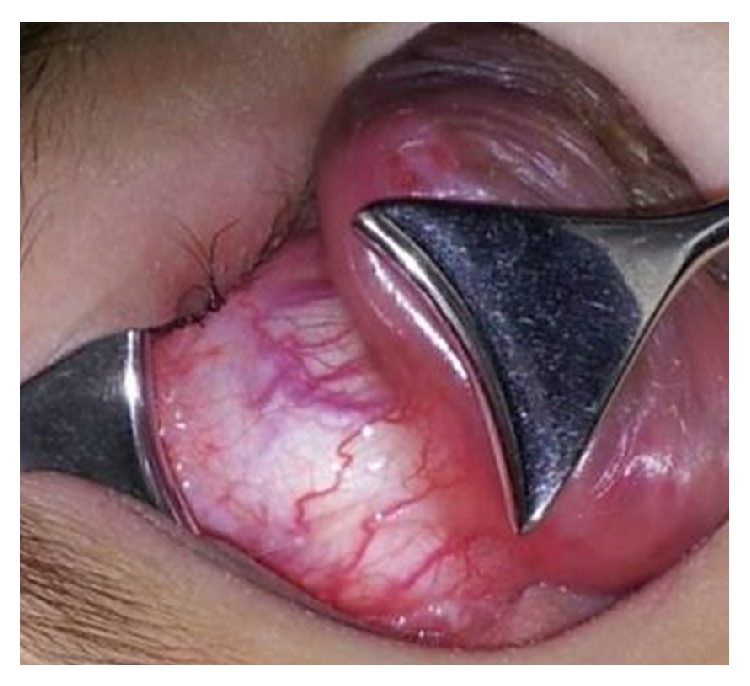
Mass adhered to the limbus and cornea but not to the rest of ocular surface of the left eye.

**Figure 6:**
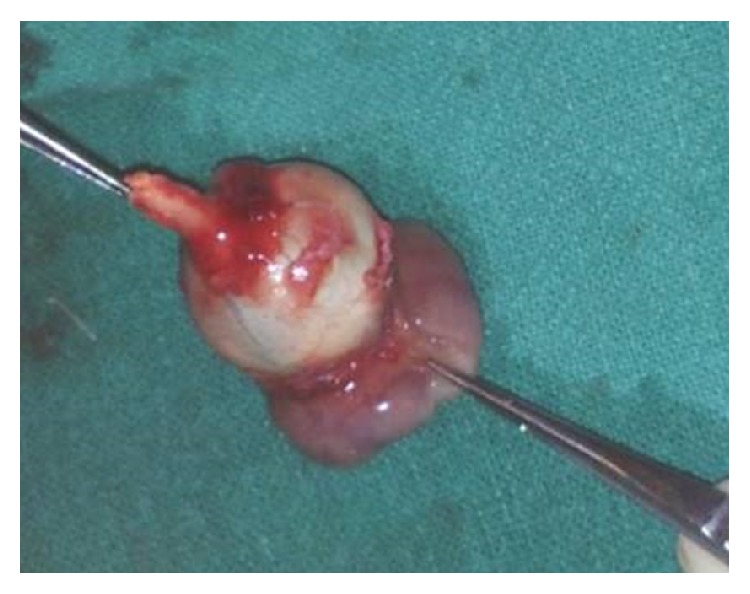
Enucleated specimen of the left eye with normal optic nerve stump.

After considering these findings, the left eye was enucleated under general anaesthesia. Gross examination of the postoperative specimen revealed a 28 × 22 × 2 mm^3^ solid greyish-white firm mass extending across the entire perilimbal area, cornea, and anterior segment (Figure 5). The optic nerve stump appeared to be normal (Figure 6).

**Figure 7:**
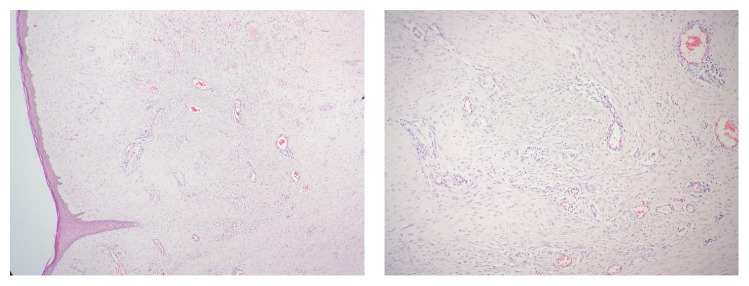
HPE of the mass showed proliferation of spindle/oval/stellate cells in fibrocollagenous stroma along with prominent vasculatures.

**Figure 8:**
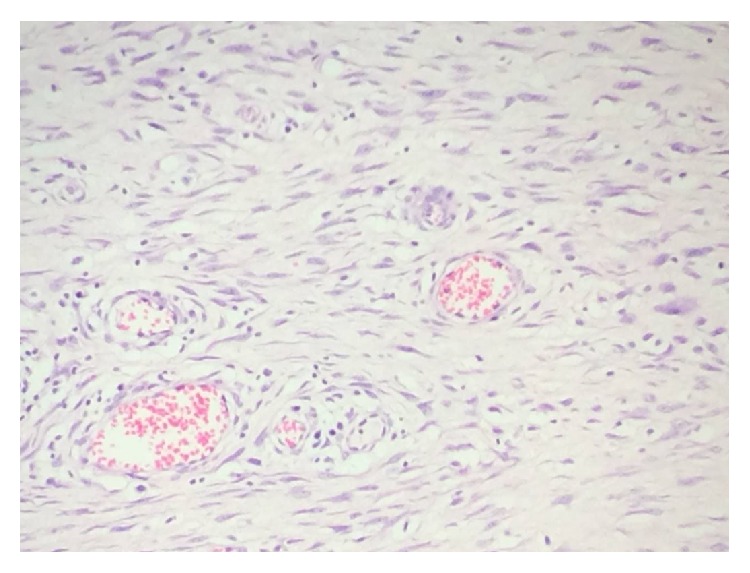
Interstitial and perivascular infiltrates by inflammatory cells.

Histopathology of the excised mass revealed tissue lined by keratinized stratified squamous epithelium. The subepithelium was composed of spindle, oval, and stellate cells in fibrocellular stroma (Figure 7). There was presence of myxoid areas in the stroma. Dilated prominent vascular channels were observed. There were interstitial infiltration and perivascular infiltration by inflammatory cells composed of lymphocytes and plasma cells (Figure 8). Mitosis and necrosis were absent and optic nerve was free of lesions. The histopathological findings were suggestive of IMT.

The postoperative period was uneventful and the child is on regular follow-up with no evidence of recurrence till date. The parents were counselled about rehabilitation of the child and use of prosthetic eye for cosmetic purpose.

Informed consent was obtained from the parents of the child for use and publication of the photographs and other related materials in scientific journal.

## 3. Discussion

IMT is a benign condition characterised by myofibroblastic spindle cells over an inflammatory background. Orbital involvement may be unilateral or bilateral and may consist of enlargement of extraocular muscles, as well as an intraconal or extraconal mass. Children and young adults are commonly affected with no sex and race predilection. We found only seven cases of ocular involvement in IMT which has been reported in literature [1, 5–10].

However, our case is the only one reported having isolated anterior segment involvement with IMT till date.

Our patient was eighteen-month-old male child, while other reported cases of IMT with ocular involvement ranged from 8 months to 50 years of age. It should be noted that most of the reported cases were male [1, 5–7].

The mode of presentation of IMT varies. Our patient presented with a mass arising from perilimbal area. The other reported cases presented with a subconjunctival mass inducing diplopia [1], a mass inferior to inferior rectus muscle with multiple neuropathy [5], painful exophthalmos [6], painless gradual diminution of vision for 2 months [9], supraorbital mass with loss of vision and phthisis for 2 years [10], and rapid progressive painless proptosis [8]. The case that was most similar to ours was an eight-month-old infant, who presented with a 5-week history of painless progressive swelling in the left upper eyelid [7].

The pathogenesis of IMT is thought to be idiopathic. Other possible causes include surgery, trauma, T and B cell lymphoma, and autoimmune reaction. IMT has been reported in association with vasculitis, inferior vena caval thrombosis and infections with mycobacterium, Epstein-Barr virus, actinomycetes, and mycoplasma [11].

CT scan and/or MR imaging of all cases, including ours, showed a mass involving the orbit [1, 5]. Multiple cranial nerve involvement (II, V1, V2, V3, and X) was only seen in the case of a fifty-year-old adult patient [5], while the CT of another case showed an intraconal mass of left orbit that was not well differentiated by the lacrimal gland [6]. The MRI orbit of one case showed a smooth lobulated mass involving the superior and lateral aspects of the right orbit with involvement of the greater wing of sphenoid bone [9]. Bone erosion was not seen in any of these cases. However, Lauwers et al. [12] reported a case of 71-year-old man with painless diplopia and proptosis of the left eye with relative afferent papillary defect (RAPD) and restricted extraocular movement. Imaging showed a left sino-orbital tumor with intracranial extension through superior orbital fissure with bone erosion and sclerotic bone reaction and this case may be the only one reported with bone invasion.

Due to the extent of the tumor, our patient was managed by enucleation. Other reported cases were treated with a combination of surgery and oral prednisolone. A ten-year-old boy underwent partial excision along with oral prednisolone [1] while fifty-year-old one received only oral prednisolone [5]. A seventeen-year-old one underwent a total removal from a lateral route of the orbit. In this case, the lacrimal gland of the same eye was also removed because of its hypertrophic appearance and hard consistency [6]. The eight-month-old infant underwent surgical debulking of the superior orbital mass through an upper eyelid crease incision [7]. The eleven-year-old girl underwent mass excision [9].

There are few cases of orbital involvement due to IMT in the surrounding structures [12, 13]. In a case reported by Chong et al. [13], a 27-year-old female presented with a 2-month history of progressive right cheek swelling and right proptosis, right hypertropia, and diplopia due to a soft tissue mass occupying the right maxillary sinus with invasion of the cheek, orbit superiorly as suggested by the CT scan. The mass was removed surgically and the histological findings were consistent with IMT. The patient was started on oral steroids but, due to continued diplopia, she was switched to oral methotrexate which led to resolution of the symptoms.

In all the orbital cases reported in literature [1, 5–9] including ours, the diagnosis of IMT was based on histopathological examination of the tumor with similar findings.

The basic components of IMT are lymphocytes, plasma cells, histiocytes, fibroblasts, and myofibroblasts which are present in variable proportions. Four basic histologic patterns [14] consist of

- dominant lymphoplasmacytic infiltrate,
- dominant lymphohistiocytic infiltrate,
- young and active myofibroblastic process,
- predominantly collagenized process with lymphocytic infiltrate.

All cases of IMT show strong positivity for vimentin, smooth muscle actin (SMA), and calponin [15].

IMTs are often characterized by anaplastic lymphoma tyrosine kinase-1 (ALK1) gene rearrangements [16]. This is detected in about 50% of soft tissue IMT with most orbital IMTs being negative [17]. The ALK1 expression is highly specific for IMT but not 100% sensitive and no clinical, morphological, or prognostic difference is found associated with ALK1 status of IMT [17, 18]. However, in our case, ALK1 was not done due to its unavailability.

At one-year follow-up, there was no recurrence of tumor seen in our patient. However, in the case of the ten-year-old [1], there was a recurrence of the tumor involving the cornea. The patient underwent radiotherapy, and no recurrence was seen till the next two years of follow-up. In the fifty-year-old, there was a recurrence of symptoms on attempted tapering of steroids [5]. In the seventeen-year-old, there was no tumor recurrence after 28 months of follow-up [6]. The parents of the eight-month-old infant declined postoperative orbital irradiation or systemic corticosteroids but no recurrence was noted after two years of follow-up [7].

## 4. Conclusion

Orbital IMT is a very rare entity which can present as an anterior segment mass even in early infancy. CT scan and MRI play crucial role in defining the extent of the tumor and deciding the treatment option while histopathological examination is important for confirming the diagnosis. Early diagnosis and treatment of orbital IMT are of paramount importance as delay can result in loss of the eye.
